# Multiscale Degradation Behavior and Moisture State-Dependent Mechanisms of Solidified Slurry Under Engineering Service Conditions

**DOI:** 10.3390/ma19204398

**Published:** 2026-10-19

**Authors:** Chuyi Yao, Feng Yu, Xin Chen, Feiyan Wu, Min Xu

**Affiliations:** 1 Institute of Foundation and Structure Technologies, Zhejiang Sci-Tech University, Hangzhou 310018, China; 2 Zhejiang Key Laboratory of Green, Digital and Intelligent (GDI) Renovation for Urban Infrastructures, Zhejiang Sci-Tech University, Hangzhou 310018, China

**Keywords:** solidified slurry, carbonation, C-S-H, CO_2_ transport, molecular dynamics

## Abstract

**Highlights:**

**What are the main findings?**

- An elevated-temperature and lower-humidity environment accelerated slurry degradation.
- Carbonation mainly attacked binding gels under limited calcium hydroxide buffering.
- Simulations suggest dry pores facilitate CO_2_ transport.

**What are the implications of the main findings?**

- The findings provide useful insights for the engineering application, durability assessment, and performance improvement of solidified slurry used in subgrade filling and related geotechnical applications.

**Abstract:**

Industrial solid waste-based composite binders offer an effective approach to soft soil stabilization and beneficial reuse of industrial solid wastes, but their long-term degradation mechanisms in complex in-service environments remain unclear. This study investigated the degradation behavior of slurry solidified with industrial solid waste-based binders under a simulated in-service environment, combining macroscale mechanical tests, carbonation degree characterization, TG-DTG, XRD, SEM, and molecular dynamics simulation to elucidate multiscale degradation mechanisms. The simulated condition significantly accelerated mechanical property deterioration and carbonation progression. Microstructural analyses revealed low calcium hydroxide (CH) content with only slight changes over time, whereas CaCO_3_ content increased, chemically bound water decreased, and the local Ca/Si ratio declined. These findings indicate that, beyond decomposition of hydration products such as AFt (ettringite), decalcification of calcium silicate hydrate (C-S-H) gel may be an important carbonation-related degradation pathway. Molecular dynamics simulations showed higher CO_2_ mobility in dry C-S-H pores, while wet pores strengthened short-range associations between CO_2_ molecules and Ca sites. These simulation results suggest a hypothetical moisture state-dependent transport-association mechanism: dry conditions may facilitate CO_2_ transport, whereas wet pores may provide more favorable local interfacial conditions for subsequent carbonation-related processes. This study provides a multiscale understanding of the deterioration of industrial solid waste-based solidified slurry under in-service conditions.

## 1. Introduction

The rapid expansion of global infrastructure has raised growing concerns about carbon emissions. The production of ordinary Portland cement (OPC) is one of the major sources of anthropogenic CO_2_ emissions worldwide, accounting for over 7% of global emissions [1,2]. According to the International Energy Agency (IEA) technology roadmap, developing low-carbon binders is one of the key pathways for the decarbonization of the cement industry [3].

In this context, industrial solid waste-based binders have been widely applied in geotechnical engineering, including slope stabilization, ground improvement, subgrade construction, and slurry solidification, owing to their favorable mechanical performance and significant environmental benefits [4]. These materials provide a promising way to reduce reliance on conventional OPC-based binders. This study employs an industrial solid waste-based composite binder independently developed by our research group [5]. The binder is composed of blast furnace slag (BFS), phosphogypsum (PG), silica fume (SF), and P.O. 42.5 ordinary Portland cement as the primary cementitious component. Sodium silicate (SS) serves as a chemical activator. Previous studies and field applications have demonstrated that, at equivalent dosages under standard curing conditions, this binder outperforms pure cement-based solidification systems in terms of mechanical performance [6]. It has been successfully applied in numerous subgrade construction and slurry solidification projects, demonstrating strong engineering applicability and considerable potential for broader use.

However, compared with ordinary Portland cement (OPC), which has a mature performance system and well-established durability-evaluation methods, existing studies have widely confirmed durability shortcomings in both special cements (e.g.,supersulfated cement (SSC) [7], calcium sulfoaluminate (CSA) cement [8]) and composite binders [9,10]. Among various durability issues, carbonation is a key cause of structural degradation in cementitious materials [8,11]. Upon diffusion into the material, CO_2_ dissolves in the pore solution and forms carbonate species, which subsequently react with calcium-bearing hydration products to precipitate CaCO_3_ [12,13]. This process lowers the pH of the pore solution and coarsens the pore network. As the primary hydration product, calcium silicate hydrate (C-S-H) governs both the mechanical properties and microstructure of cement-based materials [14,15] and also serves as a promising medium for carbon sequestration [16,17]. The carbonation rate of Portland cement is strongly influenced by its chemical composition and curing conditions [3]. Previous studies have shown that the carbonation rate of blended cement can be 2 to 4 times higher than that of OPC [18]. This is primarily attributed to the higher AFt content in blended cement hydration products, as AFt exhibits lower carbonation resistance than C-(A)-S-H gel [19,20].

In practical engineering, however, carbonation does not occur in isolation. Solidified slurry is subjected to long-term cyclic wetting and drying caused by rainfall infiltration, evaporation, and groundwater level fluctuations. Wetting and drying may also initiate and propagate microcracks [21,22,23], providing preferential pathways for CO_2_ ingress. This leads to simultaneous fluctuations in moisture state, evolution of pore structure, and damage to the cementitious skeleton within the material. In subtropical regions worldwide, the summer season is characterized by relatively high temperatures, low ambient humidity, and frequent temperature-humidity fluctuations. These conditions markedly accelerate CO_2_ diffusion, subjecting subgrade fill to a more severe coupled degradation environment. The temperature and humidity parameters adopted in this study are specifically selected on the basis of this typical climatic characteristic, with the aim of representing the actual service environment conditions of road subgrades in these climate zones.

Existing studies have mainly investigated the degradation behavior of specimens under humidity variations or under pure carbonation conditions, focusing on macroscopic mechanical properties, phase evolution, and durability indicators. A systematic understanding of the degradation mechanisms arising from the gap between field service environments and standard laboratory curing conditions remains lacking. In addition, conventional microstructural characterization methods can identify carbonation products, characterize morphological evolution, and quantify changes in phase composition. However, they mainly capture the cumulative state after exposure and cannot directly reveal the intrinsic mechanisms of interfacial reactions at the atomic scale. As a powerful tool for investigating interfacial interactions at the atomic scale, molecular dynamics (MD) simulation has been widely used to explore various nanoscale mechanisms. These include gas diffusion and adsorption [24,25,26,27,28,29] interfacial carbonation reactions [30,31], and structural changes in calcium silicate hydrate (C-S-H) [32,33,34]. MD simulations can therefore provide critical nanoscale insights for establishing a comprehensive cross-scale understanding of degradation mechanisms.

Based on these considerations, this study focuses on the performance differences in slurry solidified with industrial solid waste-based binders under standard laboratory curing conditions and in a simulated service environment. Two groups were established: a standard-curing control group and a simulated-service exposure group. The degradation behavior and underlying mechanisms were then systematically investigated. At the macroscale, unconfined compressive strength, splitting tensile strength, and carbonation analysis were conducted to evaluate mechanical deterioration and carbonation development under varying environmental conditions. At the microscale, TG-DTG, XRD, and SEM were used to analyze carbonation products, the evolution of hydration products, and microstructural damage. At the nanoscale, C-S-H was selected as a representative cementitious phase, and its nanopores were used to model transport and interactions. The lower-humidity and wet environments that may occur during service were represented by two typical pore states: dry and wet pores. Differences in CO_2_ transport and interfacial interactions were then compared. This comparison was used to reveal how pore water affects CO_2_ transport and interfacial reaction tendencies. Together, these analyses aim to clarify the degradation mechanisms of solidified slurry under a simulated service environment. It considers macroscale mechanical performance, microscale phase evolution, and nanoscale interactions. The findings provide a basis for durability assessment and service performance improvement of industrial solid waste-based solidified slurry.

## 2. Materials and Methods

### 2.1. Raw Materials and Properties

#### 2.1.1. Slurry

**Figure 1:**
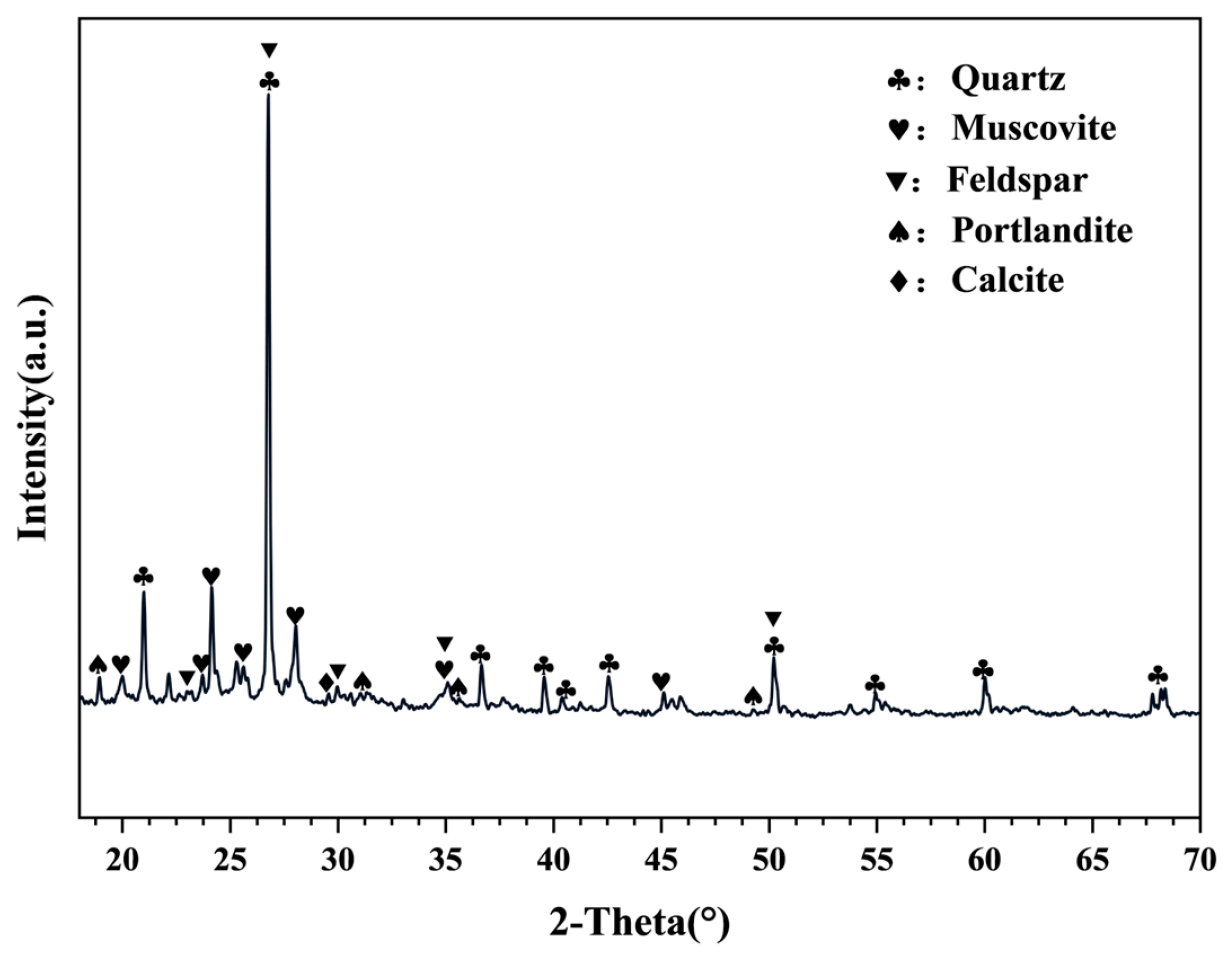
X-ray diffraction (XRD) pattern of the engineering slurry.

The test slurry was collected from a construction site in Zhejiang Province, China, and served as the target material for solidification. The basic physical properties of the test slurry are shown in Table 1. As shown in Figure 1. X-ray diffraction (XRD) analysis shows that the primary minerals are quartz, muscovite, and feldspar. Weak calcite peaks were also detected.

#### 2.1.2. Solidification Materials

The binder used in this study was prepared from BFS, PG, SS, SF, and P.O. 42.5 ordinary Portland cement.

The oxide compositions of the raw materials were determined by X-ray fluorescence (XRF) analysis. The results are shown in Table 2. The particle size distributions of the raw materials were measured using a laser particle size analyzer. The results are shown in Figure S1.

### 2.2. Testing Methods

#### 2.2.1. Specimen Preparation

The total binder content in this study was 20% by total mass of the slurry. The specimens were mixed, molded, and cured under standard conditions (20 °C, ≥95% RH) for 28 d, then divided into two groups. Group A was maintained under the same standard conditions. Group B was transferred to a ventilated environmental chamber under natural carbonation conditions, set at 35 °C and 80% RH with continuous air circulation. The overall experimental scheme is illustrated in Figure S2. These temperature and humidity parameters were selected to reflect typical daytime summer meteorological conditions in subtropical regions worldwide.

#### 2.2.2. Mechanical Properties

At the specified ages, the unconfined compressive strength and splitting tensile strength were measured in accordance with the Chinese standard “Test Method of Cement Mortar Strength” (GB/T 17671-2021 [35]). All tests were conducted using a universal testing machine. Six replicate specimens were prepared for each group to minimize random error.

#### 2.2.3. Carbonation Analysis

The degree of carbonation was characterized using carbonation depth and the carbonated area ratio. The method was based on the phenolphthalein colorimetric method specified in the Chinese standard for test methods for long-term performance and durability of concrete (GB/T 50082-2024 [36]). After splitting tensile testing, loose dust and debris were removed from the fresh fracture surfaces. A 1% phenolphthalein ethanol solution was then sprayed uniformly onto the surfaces. The specimens were kept at room temperature in the dark until the color boundary became stable and clearly visible. Images of the fracture surfaces were captured using a high-resolution camera with fixed imaging parameters. The carbonated area ratio was calculated by image analysis. Ten measurement points were uniformly selected along each fracture surface to measure the carbonation depth. The arithmetic mean of these measurements was taken as the average carbonation depth for each specimen.

#### 2.2.4. SEM, XRD, and TGA

Powder samples for microstructural characterization were prepared as follows. The samples were immersed in anhydrous ethanol for 3 days to arrest hydration. They were then dried in a vacuum oven at 40 °C for 24 h. The dried solids were subsequently ground into a fine powder and sieved through a 200-mesh sieve.

The microstructure was examined using a field-emission scanning electron microscope (FE-SEM; ZEISS Sigma 360, Jena, Germany). Fresh fracture surfaces were coated with gold to ensure adequate electrical conductivity [7].

X-ray diffraction (XRD) analysis was performed using a D8 ADVANCE diffractometer (Bruker, Germany) with Cu-Kα radiation. The 2θ scan range was 5–70, with a step size of 0.02° and a scan speed of 2°/min.

Thermogravimetric analysis (TGA) was performed using a DTG-60AH instrument (Shimadzu Corporation, Kyoto, Japan). The tests were carried out in an N_2_ atmosphere to prevent carbonation during heating [37]. The samples were heated from 20 °C to 900 °C at a constant rate of 10 °C/min. The contents of chemically bound water, calcium hydroxide, and calcium carbonate were calculated from the following equations [38] and then normalized.

(1) $$\mathrm{Bond}\mathrm{water}(\%)=\frac{W_{50}-W_{200}}{M850\;{}^{\circ}C}$$

(2) $$\mathrm{Portlandite}(\%)=(\frac{CH_{W}(M400\;{}^{\circ}C-M500\;{}^{\circ}C)\times \left(\frac{74}{18}\right)}{M850\;{}^{\circ}C})\times 100\%$$

(3) $$C_{C}(\%)=(\frac{C_{C}(M550\;{}^{\circ}C-M850\;{}^{\circ}C)\times \left(\frac{100}{44}\right)}{M850\;{}^{\circ}C})\times 100\%$$

Here, CH_w_ is the mass loss due to portlandite dehydration, Cc is the mass loss due to carbonate decomposition, and M is the mass loss of the sample at a specific temperature [39].

For SEM, XRD, and TGA analyses, one representative sample was selected from each group based on the median compressive strength of the six replicates to ensure the analyzed sample reflected the group’s average performance.

#### 2.2.5. Molecular Dynamics Simulation Methods

Based on the “realistic” cement hydrate model proposed by Pellenq et al. [40], the 11 Å tobermorite crystal structure was used as the starting model. From this, the C-S-H model with a Ca/Si ratio of 1.67 was constructed. This was achieved by removing the bridging Q^2^ silicate tetrahedra from the silicate chains [24,41]. Although the solidified slurry investigated in this study contains a complex assemblage of hydration products, C-S-H was selected as a representative Ca-bearing binding phase to probe nanoscale CO_2_ transport and interfacial association. Based on this C-S-H structure, a slit-pore model, hereafter referred to as a nanopore, was further developed. The model was used to compare the behavior of CO_2_ under different pore moisture states. Such slit-pore configurations have been widely used to study the adsorption and diffusion of small molecules in porous media [24,42].

**Figure 2:**
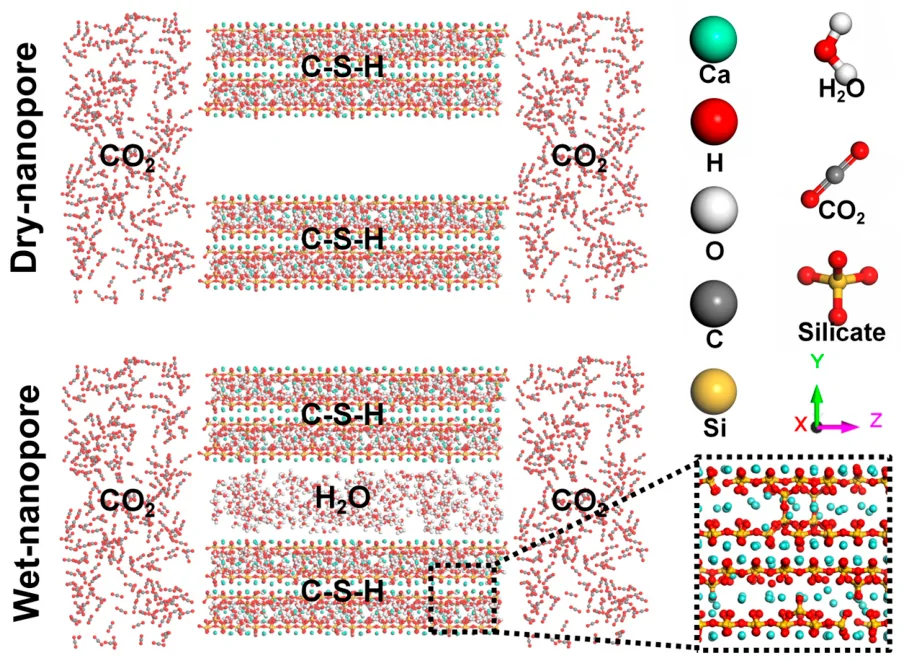
Molecular dynamics models of dry and wet C-S-H nanopores used to investigate CO_2_ transport behavior.

To systematically examine the diffusion behavior of CO_2_ within C-S-H nanopores, CO_2_ reservoir regions were introduced at both ends of the nanopores. The density of water molecules in the system was set to 0.60 g/cm^3^, representing an intermediate state between vapor-like and liquid-like water [30,31]. The pore size, defined as the interlayer spacing, was set to 14 Å. The overall configurations of the dry and wet C-S-H nanopores are shown in Figure 2. The model dimensions were 22 Å × 64 Å × 143.8 Å. All molecular dynamics simulations were performed using LAMMPS, an open-source molecular dynamics package [43].

Three-dimensional periodic boundary conditions were applied in all simulations. The ReaxFF was employed to describe interatomic interactions in C-S-H systems [32]. This force field has been widely used in multiscale simulations of carbonation-related processes in materials. The temperature was controlled using a Nosé-Hoover thermostat, and the same force-field and thermostat settings were adopted for the dry and wet nanopore systems to ensure a consistent comparison.

Before the production simulations, the system was pre-equilibrated through a two-step procedure. First, the initial configuration was optimized by energy minimization using the conjugate gradient algorithm to eliminate atomic overlaps and other physically unrealistic configurations. The minimized structure was then relaxed in the NVT ensemble at 293 K for 2 ns to obtain a stable initial configuration for the subsequent production run. The integration time step for the production simulations was set to 0.1 fs to ensure numerical stability in the ReaxFF calculations. The temperature, boundary conditions, force-field settings, and thermostat scheme were kept identical for the dry and wet nanopore systems, so that the comparison reflected the effect of the pore moisture state.

## 3. Results and Discussion

### 3.1. Strength Characteristics

**Figure 3:**
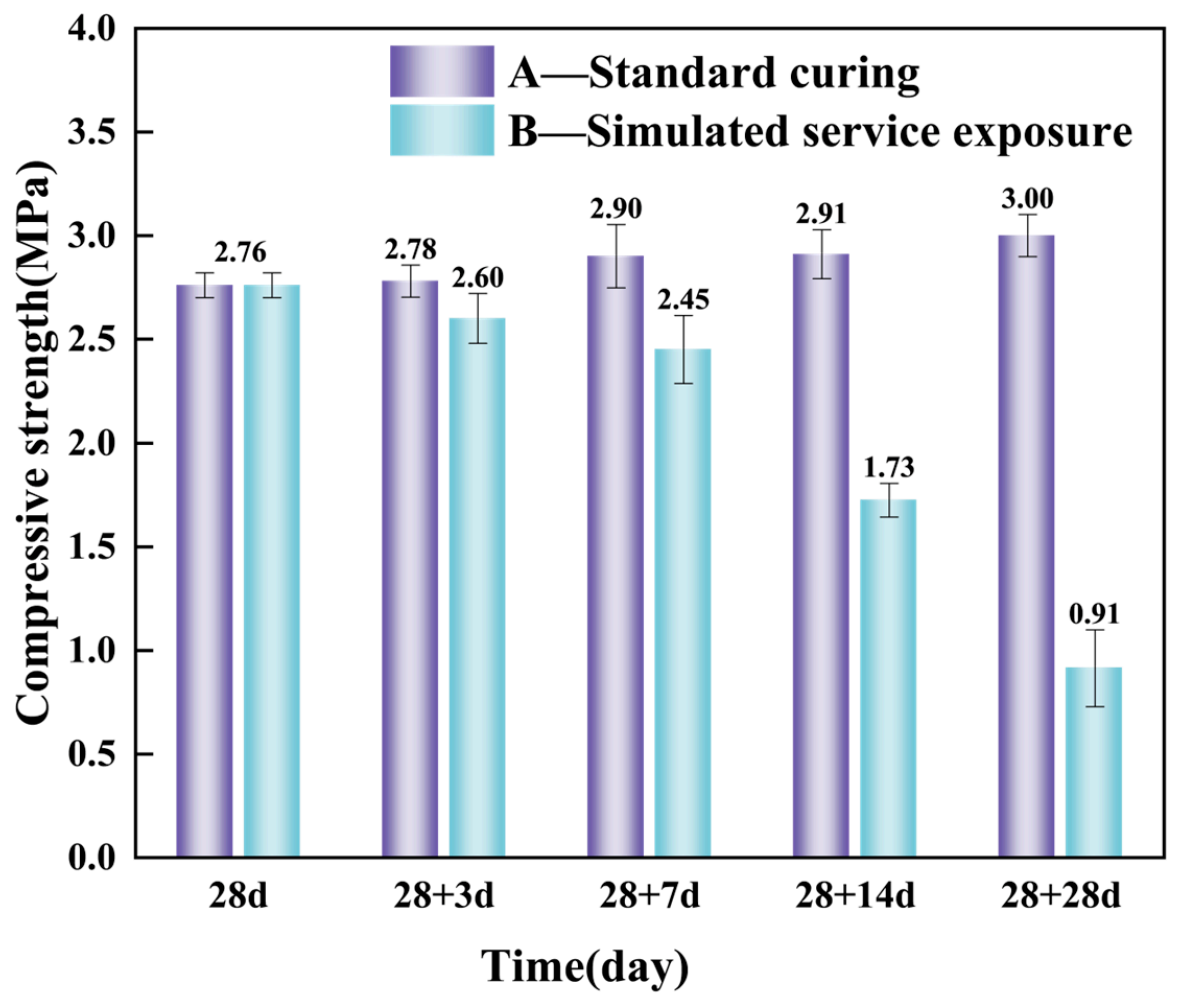
Variation in unconfined compressive strength of solidified slurry at different exposure ages after 28 days of standard curing.

Figure 3 shows the evolution of compressive strength of the specimens after the initial 28 days of standard curing. The specimens were subsequently exposed to either standard curing conditions or the simulated service environment. Six replicate specimens were tested for each condition. The mean 28-day compressive strength of the same batch of specimens, 2.76 MPa, was used as the reference value. The results show that the compressive strength of Group A, which was maintained under standard curing conditions, increased only slightly before stabilizing. This suggests that the main hydration reactions had largely proceeded after 28 days, with only limited subsequent strength gain. Continued standard curing only promoted the further hydration of a small amount of unhydrated binder particles, resulting in limited additional strength gain. In contrast, the compressive strength of Group B, which was exposed to the simulated service environment, continued to deteriorate as exposure time increased. After 28 days of exposure, corresponding to a total age of 56 days, the compressive strength decreased by 66.9% relative to the reference value. Residual strength was less than 31% of that of Group A at the same age.

This strength degradation is closely related to the hydration products formed in the solidified matrix. Compared with conventional OPC systems, the solidified matrix in this study contained a relatively large amount of AFt phases and other hydration products, which play an important role in the development of compressive strength [44]. However, AFt phases are susceptible to carbonation, and carbonation-induced decomposition of needle-like AFt crystals may weaken the load-bearing skeleton and reduce compressive strength [45]. This offers a preliminary phase-based explanation for the strength degradation observed in Group B. Therefore, the compressive strength loss of Group B is likely attributable to the coupled effects of carbonation, moisture loss, and progressive microstructural damage.

**Figure 4:**
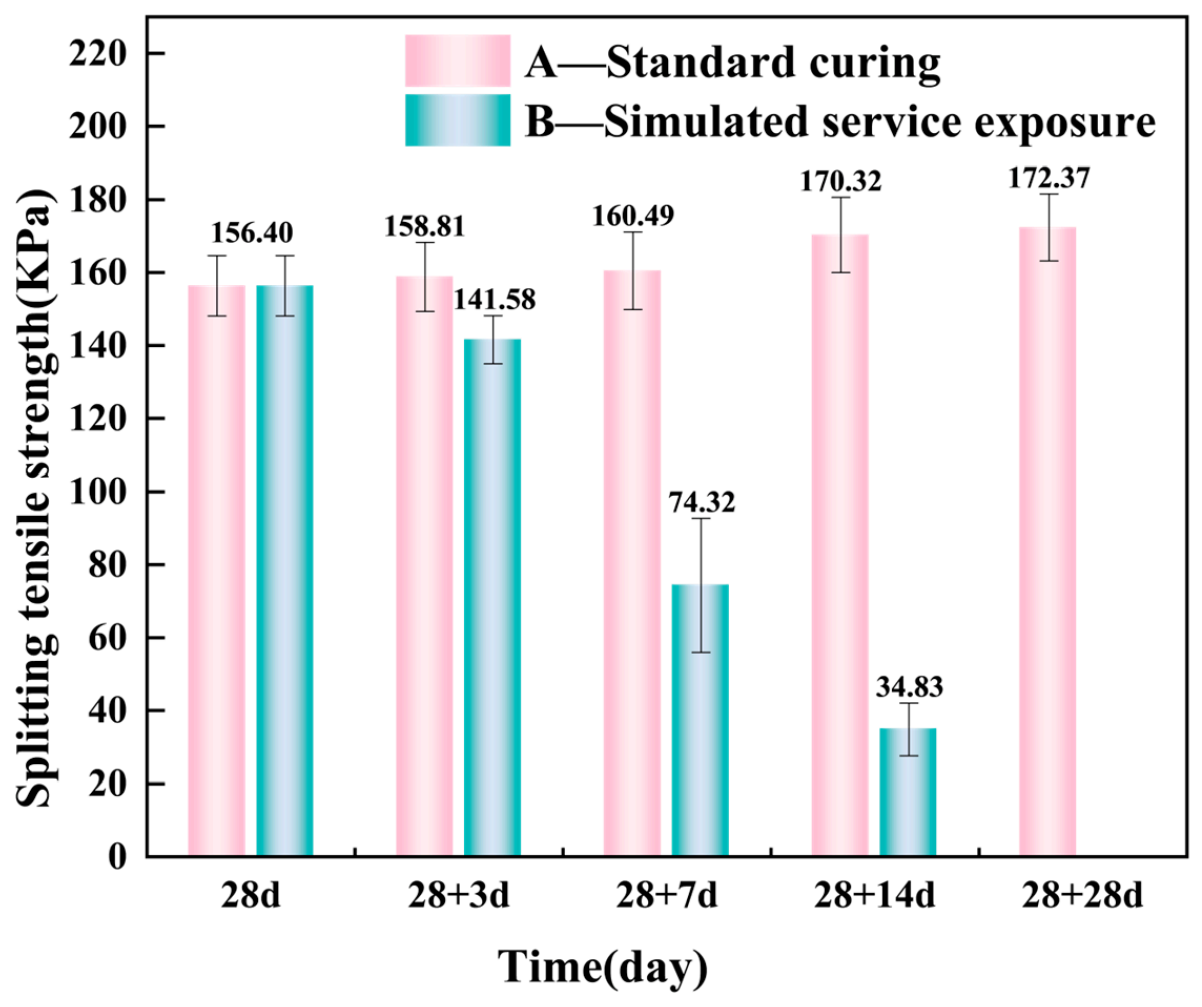
Variation in splitting tensile strength of solidified slurry at different exposure ages after 28 days of standard curing.

The splitting tensile strength of the specimens was further measured to evaluate the effects of different curing and exposure regimes on bonding performance and cracking resistance. The results are shown in Figure 4. Consistent with the evolution of compressive strength, the splitting tensile strength of Group A remained generally stable under continuous standard curing and increased only slightly with age.

The degradation trend of splitting tensile strength in Group B closely mirrored that of the compressive strength. This confirms a positive correlation between the compressive strength and splitting tensile strength of the solidified slurry. Notably, the splitting tensile strength of Group B was more sensitive to the simulated service environment. After only 7 days of exposure (total age 35 days), the splitting tensile strength had already decreased markedly. It was 53.7% lower than that of Group A at the same age. After 14 days of exposure, the reduction further increased to 79.6%. After 28 days, the specimens could no longer sustain any measurable tensile load, and no valid strength value could be recorded.

### 3.2. Carbonation Degree

**Figure 5:**
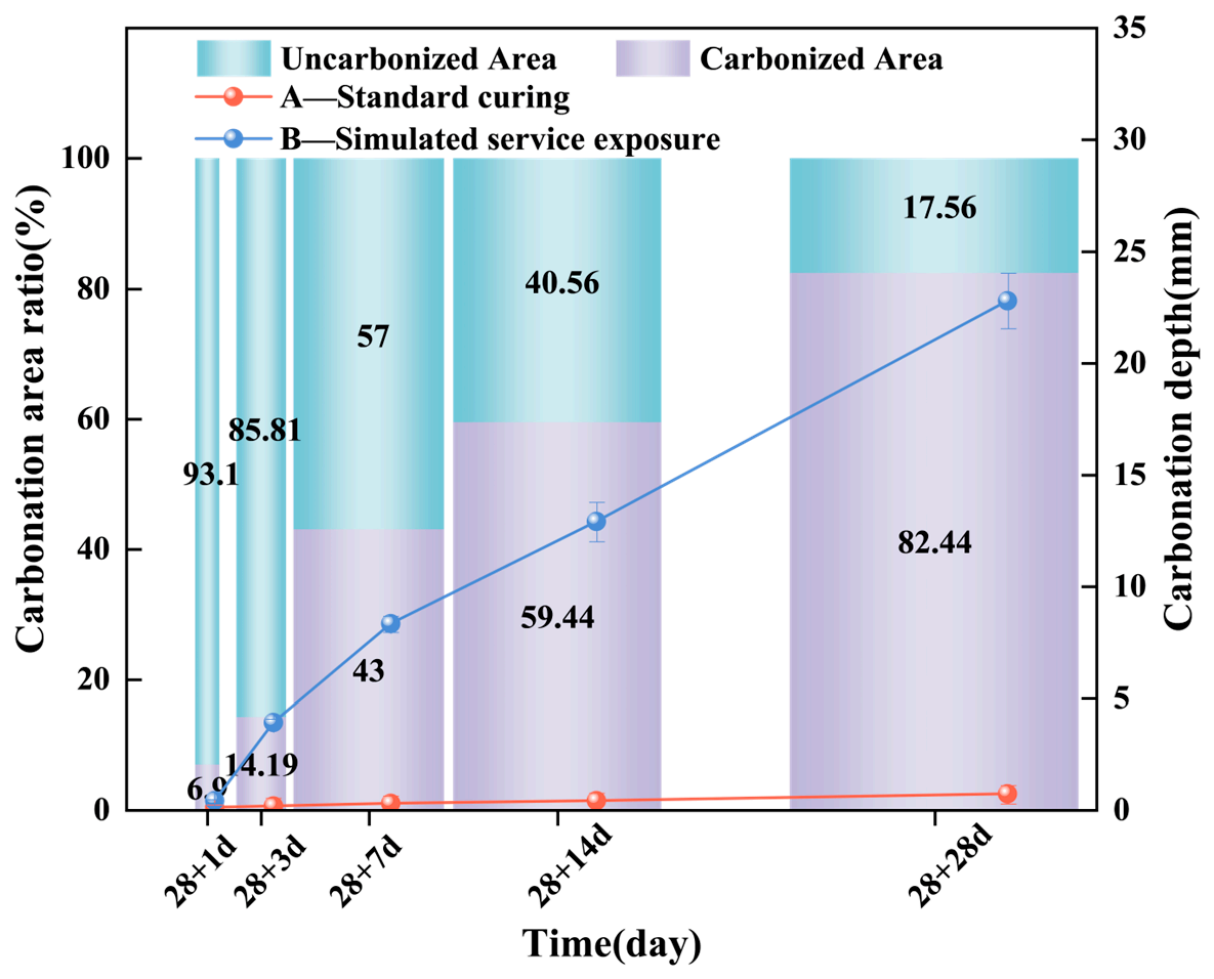
Evolution of carbonation degree with age under different curing and exposure regimes.

To further clarify the relationship between mechanical degradation and CO_2_ ingress under the simulated service environment, the degree of carbonation was characterized using carbonation depth and the carbonated area ratio. The results are shown in Figure 5. Group A was maintained under standard curing conditions and thus kept in a high-humidity, nearly saturated environment. Water-filled pores significantly hindered CO_2_ diffusion [46]. As a result, CO_2_ ingress into the matrix was severely restricted. Throughout the test period, the carbonation depth remained very low. Even at the end of the test, the depth remained below 1 mm. No appreciable carbonation development was observed.

In contrast, Group B specimens exposed to the simulated service environment exhibited rapid carbonation at an early stage, followed by a gradual slowing of the carbonation rate with prolonged exposure. Specifically, at 28 + 1 d, carbonation was confined to the near-surface region of the specimens, and the carbonated area ratio was only 6.9%. As carbonation proceeded, the carbonated area ratio increased rapidly to 43.0% at 28 + 7 d. At 28 + 28 d, only 17.56% of the cross-sectional area remained uncarbonated, and the average carbonation depth reached 22.8 mm. The carbonation rate is governed by CO_2_ diffusion, adsorption, and reaction within the pore network of the matrix. As carbonation progresses, CaCO_3_ crystals formed near the surface gradually block pore pathways [46]. This increases the resistance to CO_2_ diffusion into the matrix interior. Meanwhile, reactive phases such as C-S-H become progressively carbonated. This further restricts the progression of carbonation. Consequently, the growth rate of the carbonation depth gradually decreases with prolonged exposure.

These results indicate that the low degree of carbonation under standard curing conditions is primarily due to the inhibition of CO_2_ diffusion at near-saturated humidity. In the simulated service environment, the lower relative humidity may have produced an unsaturated pore state inside the specimens. This condition may facilitate CO_2_ transport and carbonation reactions. Meanwhile, the elevated temperature may promote moisture migration and localized drying shrinkage, further altering the pore structure and accelerating CO_2_ ingress. Because Group B was exposed to both elevated temperature and lower humidity, the performance degradation is likely the result of the coupled effect of carbonation, drying shrinkage, and pore-structure evolution. Among these factors, carbonation showed a correlation with strength loss.

### 3.3. Thermal Stability

**Figure 6:**
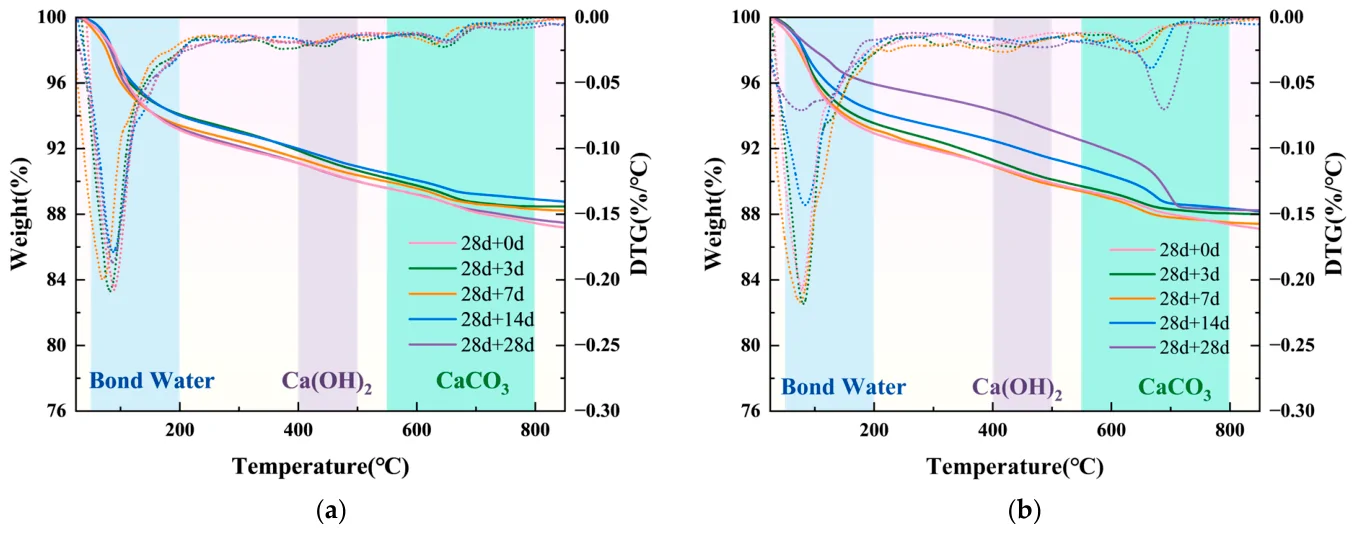
TG and DTG curves of samples under different environmental conditions. (**a**) Standard-curing group; (**b**) simulated-service group.

TG-DTG analysis was performed to quantitatively evaluate the evolution of hydration and carbonation products in the solidified slurry under different environmental conditions. As shown in Figure 6, the thermal decomposition can be divided into three main stages: (i) 50–200 °C, corresponding to the removal of chemically bound water from C-S-H and AFt; (ii) 400–500 °C, attributed to CH decomposition; and (iii) 550–800 °C, attributed to CaCO_3_ decomposition [38]. It should be noted that the calcite in this test was already present in the slurry, so the results can not fully reflect the carbonation products during the curing process. The dehydroxylation reaction of muscovite also occurs within a similar temperature range as that of CaCO_3_ [47], and these two reactions are difficult to distinguish in conventional thermogravimetric analysis. Therefore, the values calculated from these equations should be regarded as semi-quantitative estimates. Nevertheless, this method can reflect the relative change trend of carbonate content among different sample groups under unified test conditions, and still has reference value for group comparison and pattern analysis.

For Group A, the TG curves showed rapid mass loss in the range of 50–200 °C. Between 200 and 850 °C, only a slight mass loss was observed. The TG curves at different ages showed no clear separation. The corresponding DTG curves showed only a consistent peak associated with the removal of bound water. No distinct CH decomposition peak appeared over the entire temperature range. Only a very weak CaCO_3_ decomposition peak was detected between 550 and 800 °C, and it showed no significant variation with age. For Group B, the TG curves progressively diverged as exposure time increased.

**Figure 7:**
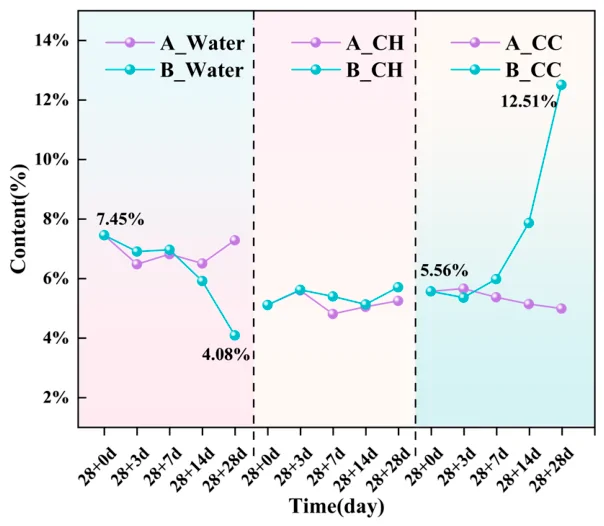
Contents of chemically bound water, calcium hydroxide, and calcium carbonate in samples under different environmental conditions. (All weight percentages are normalized to the anhydrous phase).

Based on the mass loss data in the characteristic temperature ranges of the TG-DTG curves, the relative contents of the relevant hydration and carbonation products were quantitatively calculated. The results are shown in Figure 7. The calculated CaCO_3_ content includes all calcium carbonate forms present in the system. These include amorphous calcium carbonate, metastable crystalline polymorphs such as aragonite and vaterite, and stable calcite [48,49,50,51]. Thus, the quantification is independent of the crystallinity and polymorphic form of the carbonates. It therefore gives a more comprehensive picture of the phase evolution. For Group A, the contents of chemically bound water, CH, and calcium carbonate showed no significant change during the test period. The CH content remained low, at approximately 5%. This indicates that the main hydration reactions had largely been completed after the initial 28 days of standard curing. The pozzolanic reaction of the active components had largely consumed the free CH produced by cement hydration, and no significant carbonation occurred. For Group B, the bound water content decreased by more than 45% after 28 days of exposure (total age: 56 days). The CH content still showed no significant change throughout the test period. In contrast, the CaCO_3_ content increased rapidly with exposure time. At 28 + 28 d, it reached 12.5%, representing an increase of over 125% compared with the value after standard curing.

### 3.4. XRD

**Figure 8:**
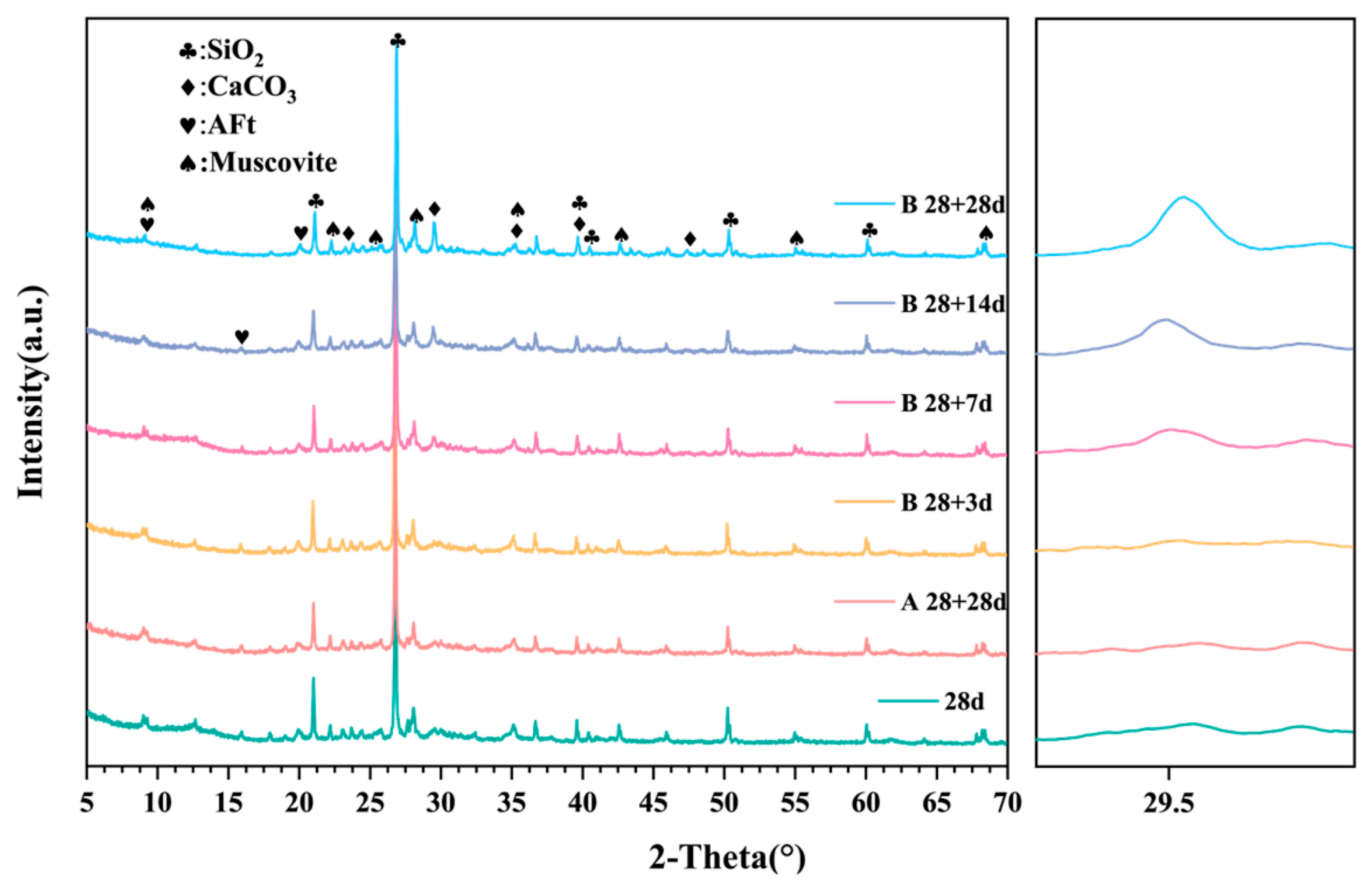
X-ray diffraction (XRD) patterns of solidified slurry under different environmental conditions.

The X-ray diffraction (XRD) patterns of the specimens are shown in Figure 8. The main detected phases included quartz and muscovite, which were inert crystalline phases introduced by the raw slurry and industrial solid waste-based binder. AFt was present as a hydration product, and calcium carbonate as a carbonation product. C-S-H gel is amorphous by nature. Its content in the solidified slurry was also relatively low. Therefore, its characteristic broad diffraction feature could not be clearly resolved by conventional XRD. Under standard curing conditions, the phase assemblage of Group A remained essentially stable. In contrast, for Group B, the characteristic diffraction peak of calcite at 29.5° [46] increased markedly with exposure time. This is also shown in the enlarged inset on the right, where the peak became increasingly sharp and intense as exposure time increased. Meanwhile, the characteristic diffraction peaks of AFt gradually decreased in intensity. These results indicate that the simulated service environment disrupted the stable cementitious phase assemblage formed after standard curing. It also induced the degradation of hydration products and the accumulation of carbonation products.

### 3.5. SEM

**Figure 9:**
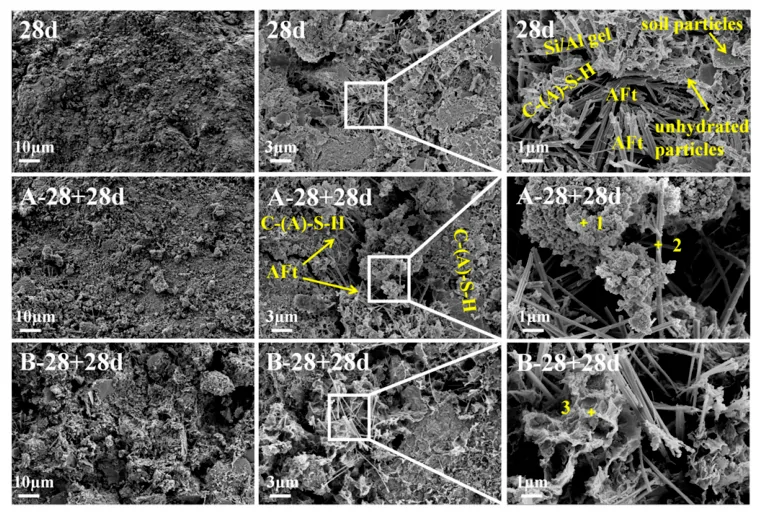
Microstructures of solidified slurry samples under different environmental conditions.

Conventional XRD has inherent limitations in accurately identifying and characterizing C-S-H gel. Therefore, scanning electron microscopy (SEM) coupled with energy-dispersive spectroscopy (EDS) point analysis was further employed to examine the microstructure and elemental composition [52]. As shown in Figure 9, the main hydration of the cementitious system had largely been completed in the 28-day reference specimen of Group A. A stable microstructure had been established. Needle-like AFt crystals formed an interlocking framework. Flocculent C-(A)-S-H gel acted as the filling and cementing phase. The hydration products tightly coated the soil particles and filled the interparticle pores. This effectively cemented the soil particles and densified the microstructure [53].

For the A-28 + 28 d specimen, which remained under standard curing to a total age of 56 days, no obvious newly formed hydration products were observed. The needle-like AFt framework and flocculent C-(A)-S-H cementing network remained relatively intact. This indicates that the standard curing conditions helped maintain the stability of the cementitious network.

After 28 days of exposure to the simulated service environment (35 °C, 80% RH), the B-28 + 28 d specimen exhibited pronounced environmentally induced microstructural degradation. Compared with Group A, the needle-like AFt phase became coarser, and the oriented interlocking skeleton was fractured and disintegrated. Meanwhile, the C-(A)-S-H gel changed from a flocculent porous structure to dense, clustered aggregates.

**Figure 10:**
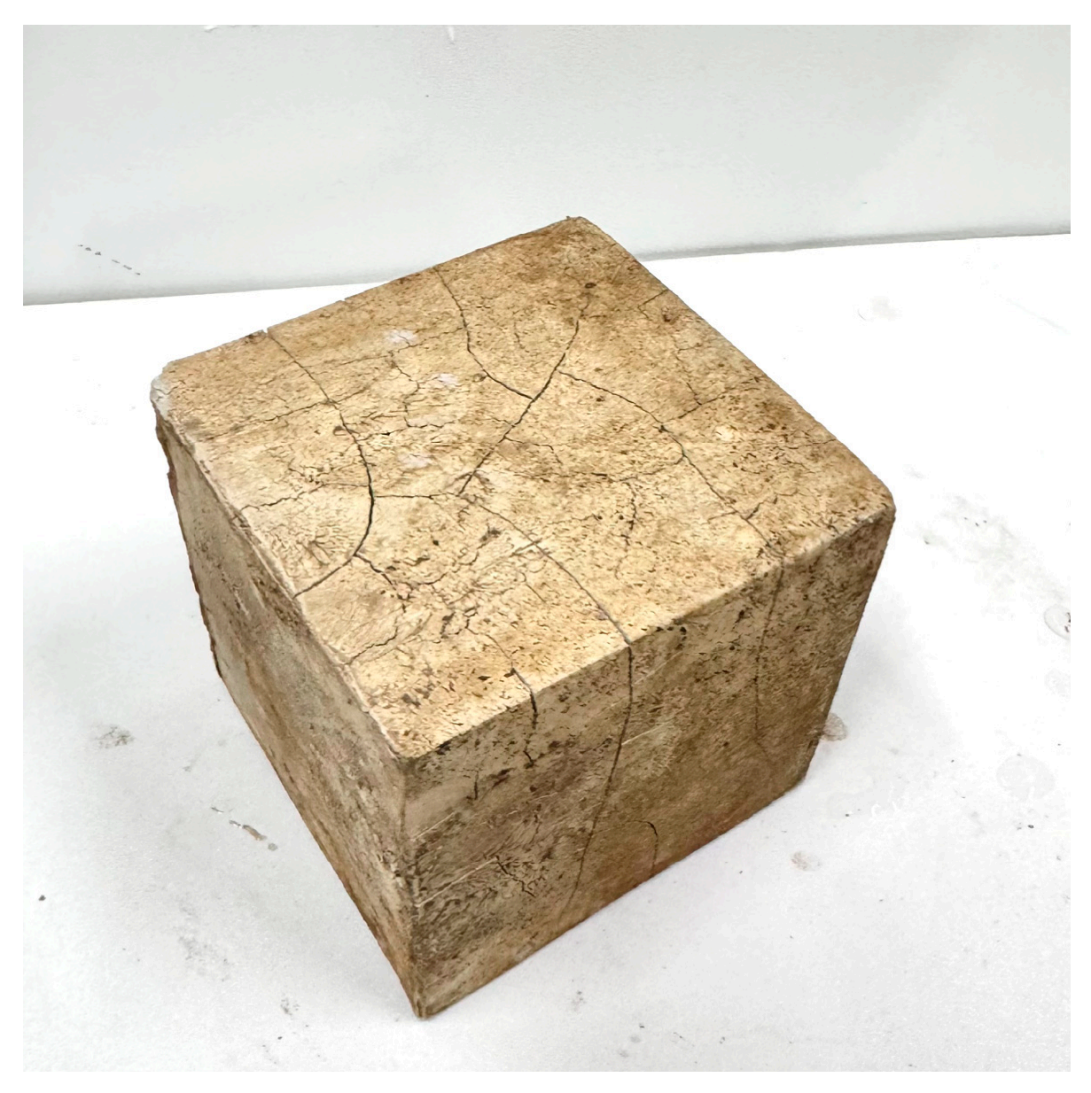
Macroscopic shrinkage crack morphology of solidified slurry after exposure to the simulated service environment.

In the carbonated regions, the C-(A)-S-H gel underwent shrinkage-induced volume changes [54]. In contrast, the volume of the uncarbonated regions remained relatively stable. This difference in volume change can induce strain in the transition zone between carbonated and uncarbonated regions. It can also alter the microstructure and increase the risk of cracking [48]. As shown in Figure 10, obvious macroscopic shrinkage cracks can be observed on the surface of the specimen exposed to the simulated service environment. These cracks provide preferential diffusion pathways for CO_2_ ingress into the matrix interior, further accelerating the carbonation process and mechanical degradation. Such shrinkage cracks may largely account for the pronounced pore coarsening, loosened microstructure, and well-developed interconnected pore networks observed in Group B.

As shown by the EDS point scan results in Table 3, the analysis further indicated that the local Ca/Si ratio of the C-(A)-S-H gel in Group B decreased, while the corresponding Al/Ca ratio increased. This indicates decalcification, namely the release of Ca from the internal structure of the C-(A)-S-H gel [55]. In addition, no obvious CaCO_3_ crystals were observed in the SEM images. This may be because the lower relative humidity during carbonation favored the formation of amorphous calcium carbonate (ACC) and metastable calcium carbonate polymorphs [11,19,56,57,58]. Their morphologies are difficult to distinguish using conventional SEM.

The combined TG-DTG, XRD, and SEM-EDS results indicate that carbonation of this industrial solid waste-based solidified system under the simulated service environment differs markedly from that of ordinary Portland cement systems. In typical OPC systems, CH preferentially dissolves [11,55,59,60] and participates in carbonation reactions. It also buffers C-S-H decalcification by maintaining a relatively high alkalinity in the pore solution [61,62,63]. Only after CH is continuously consumed and system alkalinity decreases does the decalcification-carbonation of C-S-H proceed significantly.

**Figure 11:**
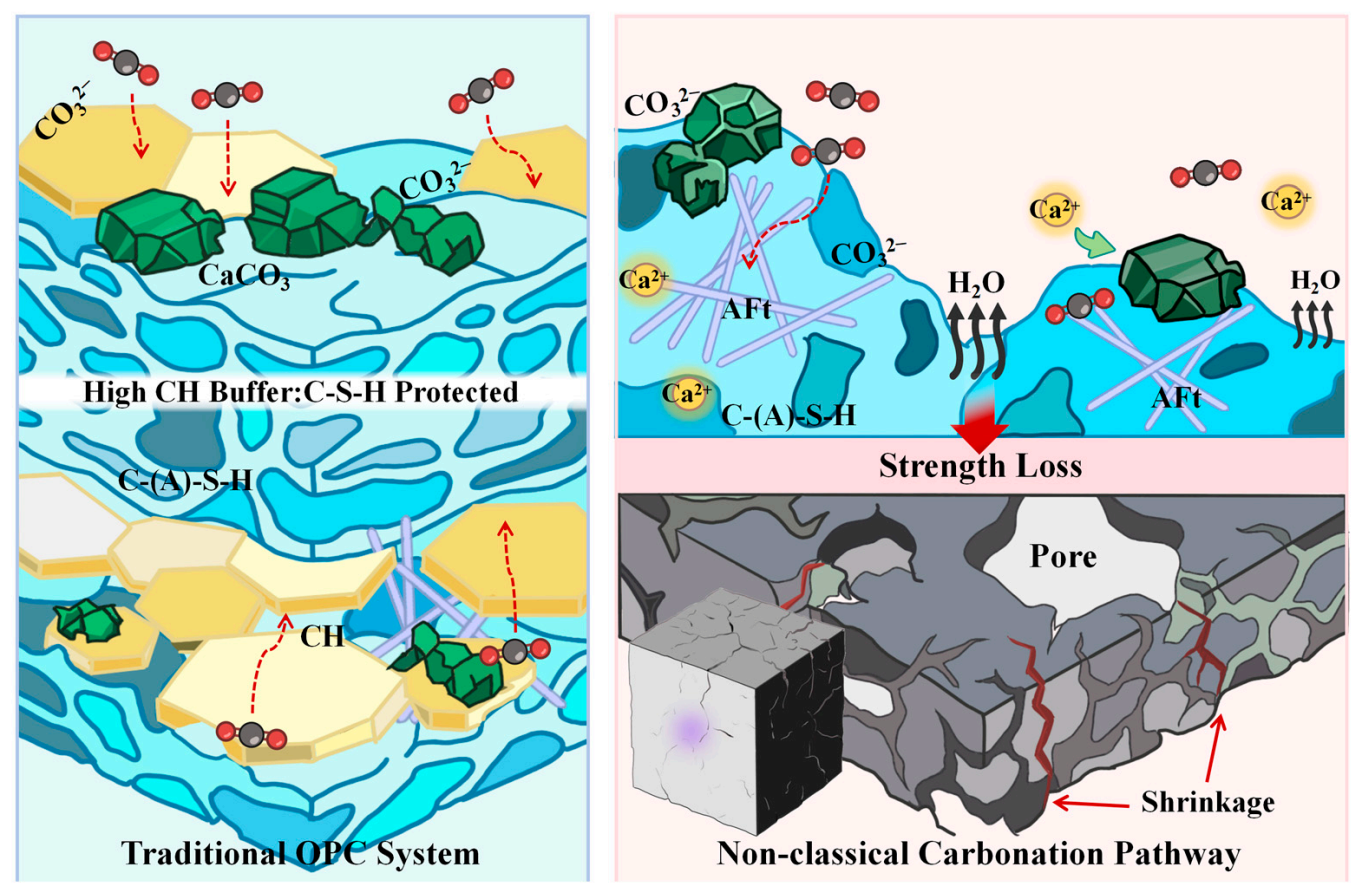
Schematic comparison of carbonation degradation pathways between the traditional OPC system and the non-classical pathway in solidified slurry.

In contrast, the CH content in the present system was low and did not decrease noticeably during exposure. Meanwhile, the CaCO_3_ content increased continuously, and the bound water decreased significantly. These changes were accompanied by a reduction in the AFt peak intensity and a local decrease in the Ca/Si ratio of C-(A)-S-H. This suggests that the newly formed calcium carbonate was not primarily derived from CH carbonation. Instead, it is more likely attributable to the decomposition of calcium-containing hydration products such as C-(A)-S-H and AFt. The release of Ca^2+^ from within the C-S-H layers leads to structural collapse [34]. This process may also cause the release of bound water and shrinkage of the binding phase, ultimately resulting in disruption of the cementitious network, pore coarsening, and crack propagation. This direct carbonation pathway of the cementitious phases under low-CH-buffering conditions is likely an important microscopic reason for the rapid strength loss and durability degradation of Group B. The corresponding mechanism is illustrated in Figure 11.

### 3.6. Molecular Dynamics Simulation

It should be noted that the present tobermorite-based C-S-H model, with a fixed Ca/Si ratio of 1.67 and no Al substitution, is an idealized representation of the real C-(A)-S-H gel. While this may introduce quantitative differences in absolute property values, the tobermorite 14 Å structure is a commonly used reference model for C-S-H atomistic studies, and the mechanistic insights and qualitative trends obtained here are expected to remain valid.

#### 3.6.1. Differences in CO_2_ Transport Behavior

The mean squared displacement (MSD) is a key parameter for analyzing the diffusion behavior of CO_2_ molecules in C-S-H nanopores. In confined spaces, molecular diffusion is anisotropic. Molecular motion along the y direction, which is perpendicular to the solid surface, is geometrically constrained by the upper and lower pore walls [27]. Therefore, the MSD in the y direction, denoted as MSDy, eventually converges to a constant value governed by the pore width.

For this reason, only the two-dimensional MSD in the x and z directions was used to calculate the diffusion coefficient. This approach more accurately reflects the in-plane diffusion behavior of molecules within the nanopore. The diffusion coefficient is expressed as follows:

(4) $$D=\frac{1}{2N}\underset{t\to \infty }{\lim}\frac{MSD_{\mathrm{xz}}(t)}{t}$$

**Figure 12:**
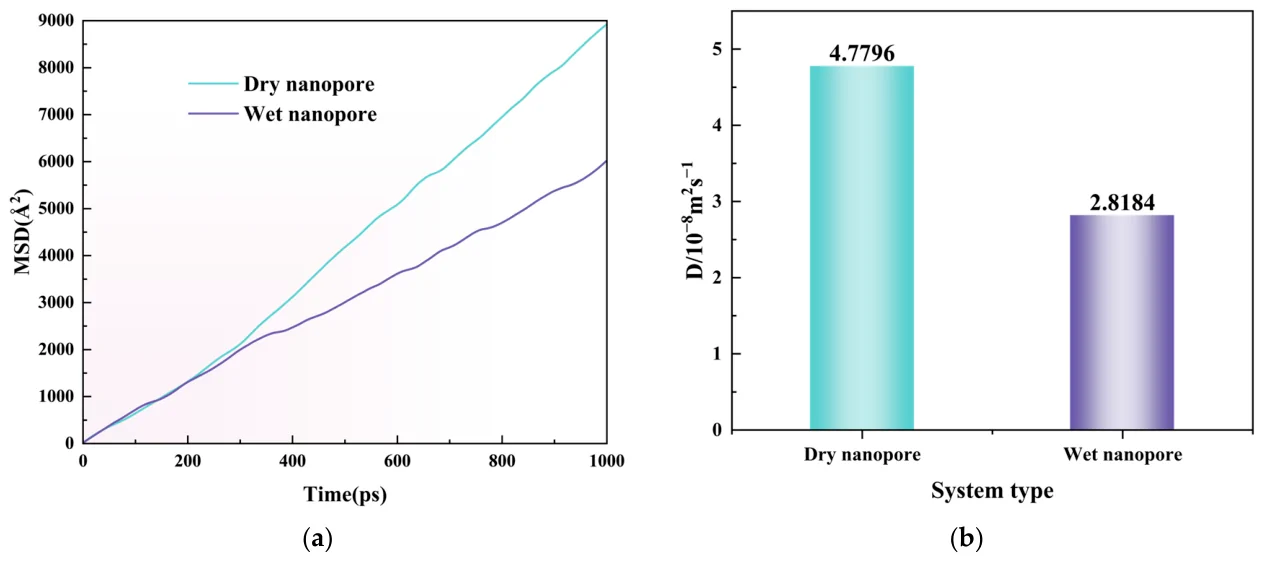
Comparison of CO_2_ diffusion behavior in dry and wet C-S-H nanopores. (**a**) Mean squared displacement (MSD) curves of CO_2_ molecules in dry and wet C-S-H nanopores; (**b**) Calculated self-diffusion coefficients of CO_2_.

Figure 12 compares the MSD curves of CO_2_ molecules in dry and wet C-S-H nanopores. The results show that CO_2_ diffusion in C-S-H nanopores depends strongly on the pore moisture state. At the initial stage, the MSD curves in the two systems were close to each other, indicating that CO_2_ motion was mainly governed by local thermal motion and short-range collisions before long-range diffusion became dominant. With increasing simulation time, the MSD of CO_2_ in the dry nanopore became progressively higher than that in the wet nanopore, and the difference between the two curves gradually increased. The diffusion coefficient of CO_2_ in the wet C-S-H nanopore was approximately 41.0% lower than that in the dry nanopore. This result indicates that the presence of confined water reduces the mobility of CO_2_ in the 14 Å C-S-H nanopore considered in this study. Water molecules occupy part of the accessible pore space and may form adsorbed water layers near the C-S-H surfaces, thereby increasing the frequency of CO_2_–water and CO_2_–surface interactions. Overall, under the present model conditions, the dry pore provides a more favorable pathway for rapid CO_2_ transport, whereas the wet pore restricts CO_2_ mobility to a certain extent.

**Figure 13:**
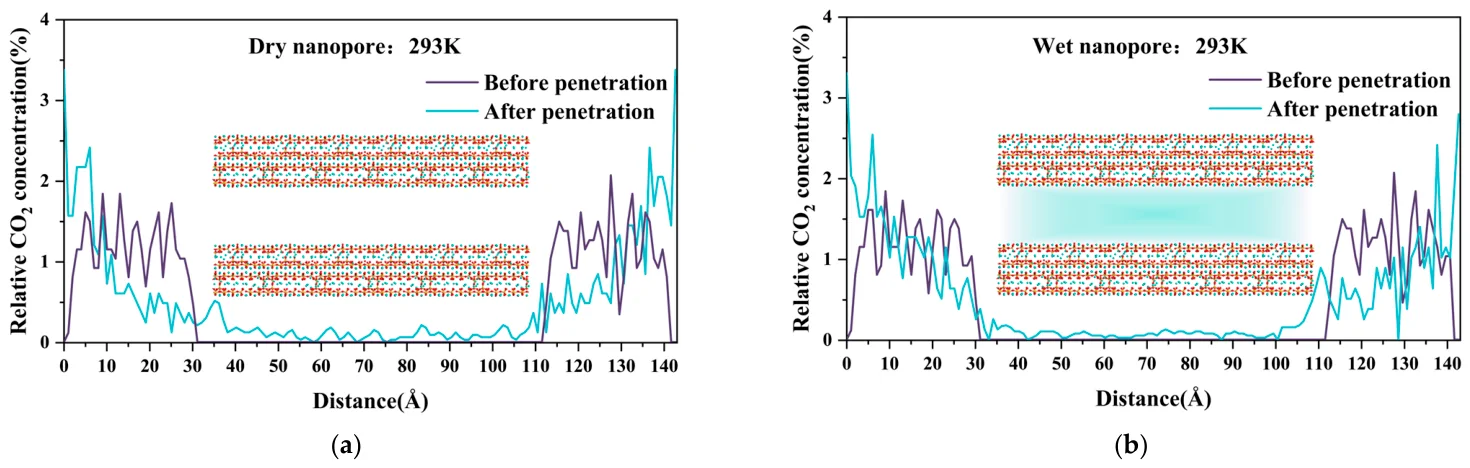
Relative concentration distributions of CO_2_ in dry and wet C-S-H nanopores before and after permeation. (**a**) Dry nanopore; (**b**) wet nanopore.

Figure 13 shows the relative concentration profiles of CO_2_ along the axial direction of the dry and wet C-S-H nanopores before and after permeation at 293 K. In this study, the z-direction regions of 0–35 Å and 108.8–143.8 Å were defined as CO_2_ reservoirs, while the region of 35–108.8 Å was defined as the C-S-H nanopore. In the initial configuration, CO_2_ molecules were placed in the two reservoirs, and the relative CO_2_ concentration in the central nanopore region was zero. As diffusion proceeded, CO_2_ molecules gradually entered the nanopore, leading to an increase in the CO_2_ concentration within the pore region. In the dry pore, the absence of confined water eliminated water-induced steric hindrance. This provided a low-resistance gas-phase diffusion pathway for CO_2_. Therefore, after diffusion equilibrium, the relative CO_2_ concentration in the dry nanopore was higher than that in the wet pore. Water molecules reduce the efficiency of CO_2_ diffusion and exchange by modifying the pore-water structure. Notably, this phenomenon is more severe in the 14 Å C-S-H nanopore than in larger pores [31].

#### 3.6.2. Analysis of Interatomic Interactions

The radial distribution function (RDF) is a key statistical tool for characterizing the spatial arrangement and local correlations of particles at the atomic scale. The RDF is defined as follows:

(5) $$g(r)=\frac{\frac{\mathrm{dN}}{4\pi r^{2}\mathrm{dr}}}{\rho }$$

where N is the total number of atoms considered, ρ is the average atomic number density, and r is the radial distance between two atoms. By integrating the RDF over a selected distance range, the coordination number (CN) can be obtained:

(6) $$n(r)=4\pi \int_{r}^{r+\mathrm{dr}}\rho r^{2}g(r)\mathrm{dr}$$

In this study, atom pairs with clear physical relevance to CO_2_ transport and interfacial association were selected for discussion. These include contacts between CO_2_ oxygen atoms (O_c_) and Ca sites in C-S-H, the short-range associations involving water molecules in wet nanopores, and atom pairs associated with the local C-S-H framework. These RDFs were used to evaluate how the pore moisture state affects local spatial correlations near the C-S-H surface.

**Figure 14:**
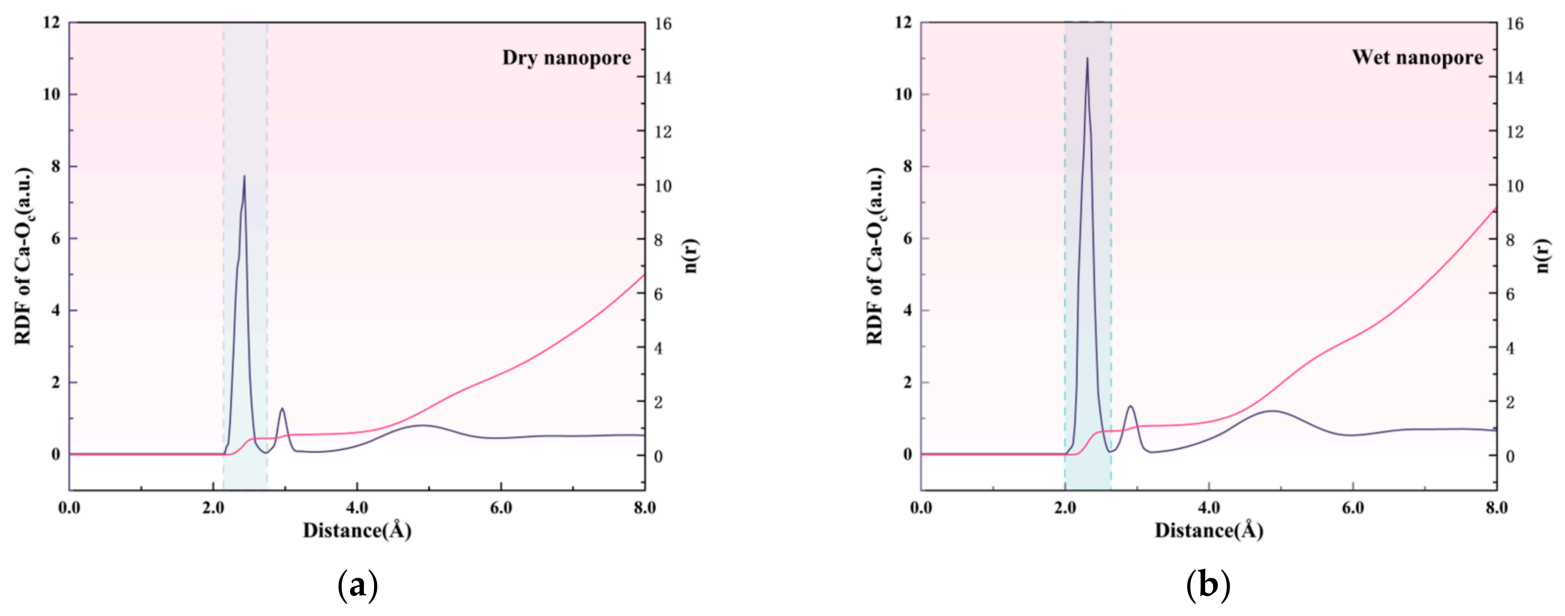
RDF characteristics between Ca atoms and CO_2_ oxygen atoms in dry and wet C-S-H nanopores. (**a**) Dry nanopore; (**b**) Wet nanopore.

Figure 14 compares the RDFs between Ca atoms in C-S-H and CO_2_ oxygen atoms in dry and wet C-S-H nanopores. The two systems exhibit similar peak distributions. A pronounced first peak appeared at approximately 2.3–2.4 Å in both cases, indicating a short-range spatial correlation between CO_2_ oxygen atoms and Ca sites on the C-S-H surface. The broader and weaker peaks at longer distances reflected longer-range spatial correlations, with no obvious difference between the dry and wet systems. This suggests that the pore moisture state mainly affects the short-range Ca–O_c_ association.

The first-peak intensity increased from 7.73 in the dry nanopore to 11.01 in the wet nanopore, corresponding to an increase of 42.4%. The coordination number from integration of the first peak also increased from 0.62 to 0.88. This indicates that water molecules promote the incorporation of CO_2_ into the first coordination shell of Ca sites, thereby increasing the direct coordination number. The underlying microscopic mechanism can be explained as follows. When water molecules coordinate with surface Ca sites, they modify the original coordination environment formed by silicate groups and surface oxygen atoms. This hydration process may enhance the local dynamics of Ca sites, thereby facilitating coordination exchange. Meanwhile, interfacial water can promote CO_2_ accumulation near the surface through CO_2_–water and CO_2_–surface interactions.

A similar enhancement of the Ca–O_c_ RDF peak in wet C-S-H nanopores was reported by Zhu et al. [31] and attributed to a bicarbonate-mediated pathway. Here, we provide a complementary structural interpretation: confined water may modulate the local coordination environment of surface Ca sites, thereby increasing the probability of close Ca–O_c_ contacts.

#### 3.6.3. Water-Mediated Short-Range Interfacial Correlations

**Figure 15:**
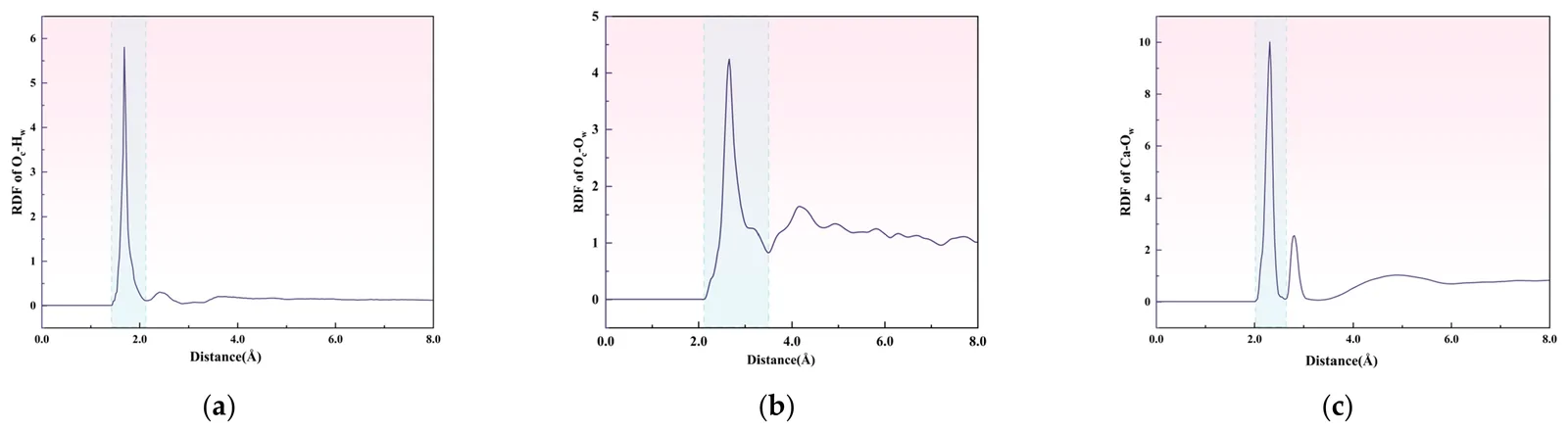
RDF characteristics of water-mediated CO_2_-C-S-H interfacial interactions in wet C-S-H nanopores. (**a**) Radial distribution function between CO_2_ oxygen atoms and water hydrogen atoms (O_c_–H_w_); (**b**) Radial distribution function between CO_2_ oxygen atoms and water oxygen atoms (O_c_–O_w_); (**c**) Radial distribution function between Ca sites on the C-S-H surface and water oxygen atoms (Ca–O_w_).

To clarify the structural origin of the enhanced Ca–CO_2_ association in wet nanopores, RDFs of three water-related atom pairs were analyzed, as shown in Figure 15. These correspond to the hydrogen bonds between CO_2_ oxygen and water hydrogen(O_c_–H_w_), the short-range oxygen-oxygen correlation between CO_2_ oxygen and water oxygen (O_c_–O_w_), and the coordination between water oxygen and Ca sites on the C-S-H surface(Ca–O_w_).

In wet C-S-H nanopores, the RDF for the Ca–O_w_ pair exhibited a sharp first peak at 2.21 Å. This indicates a pronounced short-range correlation between O_w_ and Ca. This observation confirms that water molecules do not merely fill the free pore space. Instead, they approach surface Ca sites and participate in their local hydration environment. This provides the structural prerequisite for water molecules to modify the local coordination environment of surface Ca sites.

Meanwhile, the RDF for the O_c_–O_w_ pair showed a clear first peak at 2.66 Å. The O_c_–H_w_ pair exhibited a short-range peak at 1.69 Å, suggesting hydrogen-bond-related association between CO_2_ oxygen atoms and water hydrogen atoms. These water-related correlations help explain the enhanced Ca–O_c_ association observed in Figure 14.

**Figure 16:**
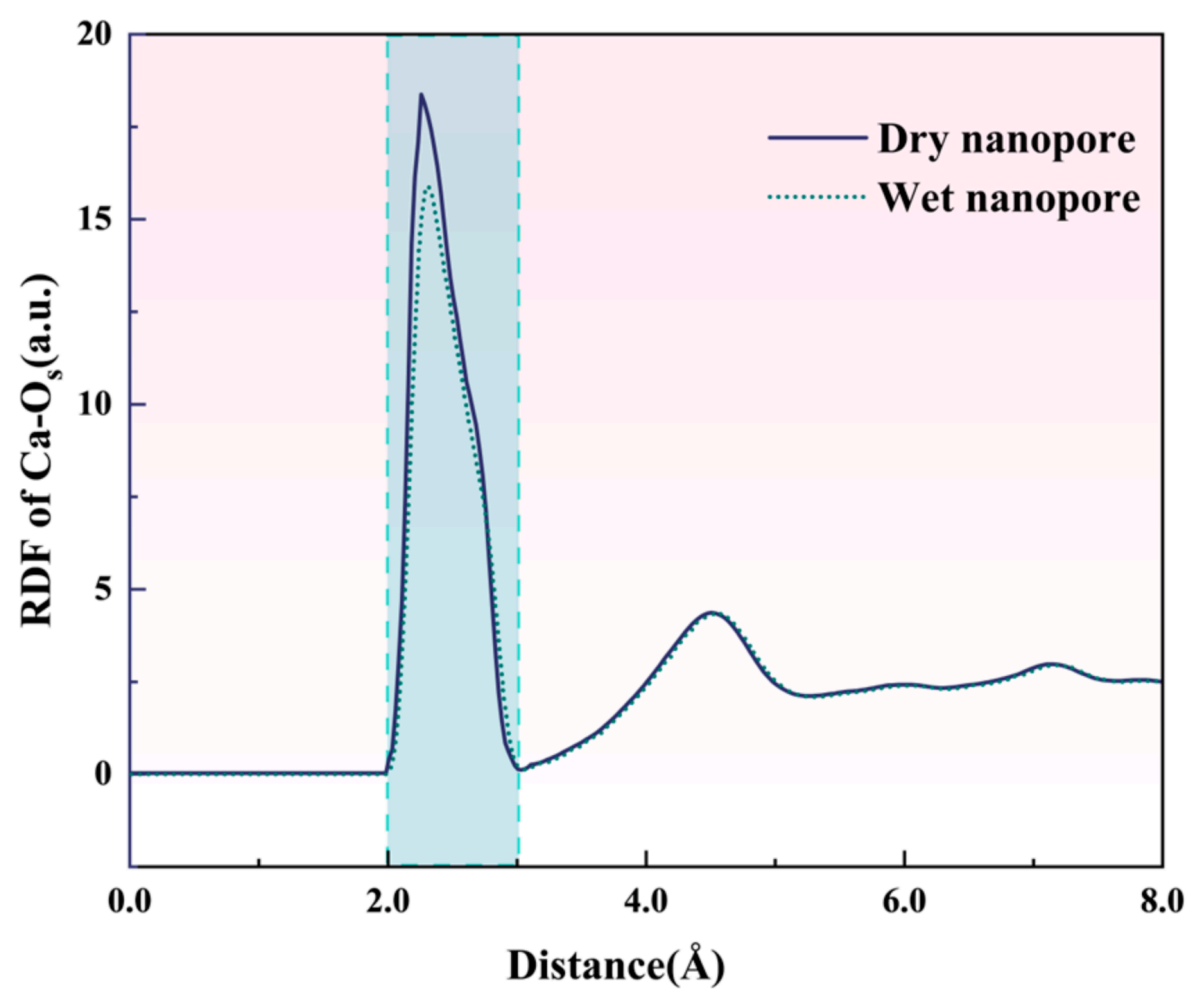
RDF response of local Ca–O_s_ coordination in dry and wet C-S-H nanopores.

Figure 16 compares the RDFs between Ca atoms and framework oxygen atoms (O_s_) in C-S-H nanopores under dry and wet conditions. Both systems showed a sharp first peak at 2.31 Å. This peak corresponds to the short-range association between Ca sites and neighboring oxygen atoms in the silicate framework of C-S-H. In the dry nanopore, the first peak was higher and narrower than that in the wet nanopore, indicating a more pronounced and ordered local Ca–O_s_ correlation. Both systems showed a second peak at approximately 4.5 Å, with similar intensity, and the curves nearly overlapped in the long-range region above 6.0 Å. These results indicate that the presence of water mainly affects the first coordination shell of surface Ca sites on the C-S-H surface. Under wet conditions, water molecules coordinate with Ca sites and partially replace framework oxygen atoms in the coordination shell. This weakens the local Ca–O_s_ correlation and slightly distorts the coordination environment. This water-induced perturbation may increase the local coordination flexibility of surface Ca sites. Previous studies have shown that the dissolution free energy of surface Ca sites decreases with decreasing initial coordination number [32]. Therefore, the weakened Ca–O_s_ correlation observed in the wet nanopore may make surface Ca sites more accessible to incoming CO_2_ molecules. This is consistent with the stronger Ca–O_c_ correlation and higher coordination number observed in wet C-S-H nanopores.

Overall, these results provide a structural explanation for the enhanced reactivity of Ca sites on the C-S-H surface under wet conditions. In real amorphous C-S-H, intrinsic atomic-scale surface defects may further amplify the heterogeneity of the coordination environment. The average coordination number of surface Ca sites is markedly lower than that in crystalline C-S-H. This makes them more susceptible to decalcification-induced degradation [32].

These simulation results suggest that during service conditions involving variations in pore moisture state, CO_2_ migration and local interfacial association may be controlled by different mechanisms.

Under the modeled dry-pore conditions, CO_2_ exhibits higher mobility, which facilitates its migration into C-S-H nanopores. In contrast, under the modeled wet-pore conditions, CO_2_ migration was restricted, but the short-range Ca–O_c_ association near the C-S-H surface was enhanced. Based on these simulation findings, it is hypothesized that the variation between dry and wet states during engineering service may follow a stage-dependent transport-reaction mechanism: dry conditions may facilitate CO_2_ transport, whereas wet pore conditions may provide a more favorable local interfacial environment for subsequent carbonation-related processes. This hypothesis remains to be verified by experiments with controlled moisture levels.

## 4. Conclusions

This study focused on the differences between standard laboratory curing conditions and the simulated service environment. It systematically investigated the degradation behavior and mechanisms of industrial solid waste-based solidified slurry at the macro-, micro-, and nanoscale. The main conclusions are as follows:

Compared with standard curing conditions, the simulated service environment of 35 °C and 80% relative humidity led to pronounced mechanical degradation and clear carbonation development. After 28 days of exposure, the compressive strength of the simulated-service group decreased by 66.9%, and the splitting tensile strength was severely reduced. The average carbonation depth, determined by phenolphthalein staining, was 22.8 mm. In contrast, the specimens under standard curing conditions maintained nearly stable strength, and the carbonation depth was less than 1 mm at the same age. These results indicate that the performance observed under standard curing conditions does not fully reflect the service stability of the solidified slurry in an unsaturated hygrothermal environment.

The microscale results show that the CH content in this system was low and remained nearly unchanged throughout the test period. These changes were accompanied by degradation of AFt phases, morphological deterioration of C-(A)-S-H gel, and a local decrease in the Ca/Si ratio. The degradation appears not to follow the classical pathway of preferential CH carbonation commonly observed in plain OPC systems. Instead, it appears to be dominated by decalcification and decomposition of C-(A)-S-H gel, accompanied by degradation of AFt phases. This process damaged the cementitious network, coarsened the pore structure, and induced microcracking. These changes ultimately governed the macroscopic loss of strength.

Molecular dynamics simulations further indicated that CO_2_ transport and local interfacial association in C-S-H nanopores are strongly affected by pore moisture state. Dry C-S-H nanopores favored rapid CO_2_ diffusion. This indicates that CO_2_ ingress is more likely during the drying stage of solidified slurry. In contrast, wet C-S-H nanopores restricted long-range CO_2_ migration but enhanced the short-range Ca-O_c_ association near the C-S-H surface. Therefore, the variation between dry and wet states during engineering service may follow a stage-dependent transport-reaction mechanism: dry conditions may facilitate CO_2_ transport, whereas wet pore conditions may provide a more favorable local interfacial environment for subsequent carbonation-related processes. This offers a potential nanoscale explanation for the observed development and degradation of cementitious phases during carbonation.

From an engineering perspective, cracking is a key factor affecting the durability of solidified slurry. Yoo et al. [64] reported that fibers can effectively control crack width and restrict transport paths through fiber bridging, and that fiber type and dosage significantly influence the effectiveness of crack control. Therefore, incorporating an appropriate amount of fibers into solidified slurry to inhibit shrinkage cracking and slow down CO_2_ ingress is a potential approach to improving long-term durability.

Future work will further investigate temperature and humidity fluctuations, variations in CO_2_ concentration, pore-size distribution, and moisture migration under field service conditions. This will help establish intrinsic correlations and quantitative links across different length scales, and explore the effect of fiber content on crack development control.

## Figures and Tables

**Table 1:** Basic physical properties of the slurry.

Sludge Properties	Values
Moisture Content/%	66.86
Cohesion/kPa	10
Internal Friction Angle/°	27.2
Liquid Limit/%	26.8
Plastic Limit/%	18.5
Compression Modulus/MPa	10.3

**Table 2:** Oxide compositions of the raw materials.

Materials	CaO	SiO_2_	Al_2_O_3_	MgO	SO_3_	Fe_2_O_3_	TiO_2_	Na_2_O
BFS	67.56	12.24	9.01	4.58	0.76	0.98	2.00	-
PG	66.85	3.49	0.71	-	25.34	1.55	-	-
SS	0.36	77.26	0.40	-	-	0.20	-	21.02
SF	1.13	97.46	0.39	-	-	0.30	-	-
P.O. 42.5	51.42	24.99	8.26	3.71	2.51	4.03	-	-

**Table 3:** EDS elemental analysis results of typical hydration products in solidified slurry.

Number Mineral	1 C-(A)-S-H	2 AFt	3 C-(A)-S-H
Al	5.11	7.51	5.56
Si	8.87	13.63	12.9
S	4.25	4.48	3.74
Ca	16.25	39.1	16.78
Ca/Si	1.83	2.86	1.3

## Data Availability

The original contributions presented in this study are included in the article/Supplementary Material. Further inquiries can be directed to the corresponding author.

## Supplementary Materials

The following supporting information can be downloaded at https://www.mdpi.com/article/10.3390/ma19204398/s1, Figure S1: Particle size distributions of the raw materials; Figure S2: Schematic illustration of specimen preparation and experimental design under different service conditions.

## Abbreviations

The following abbreviations are used in this manuscript:

CH	Calcium Hydroxide
AFt	Ettringite
C-S-H	Calcium silicate hydrate
OPC	Ordinary Portland cement
BFS	Blast furnace slag
PG	Phosphogypsum
SF	Silica fume
SS	Sodium silicate
SSC	Supersulfated cement
CSA	Calcium sulfoaluminate
