# Supplementary material for: Multiscale Degradation Behavior and Moisture State-Dependent Mechanisms of Solidified Slurry Under Engineering Service Conditions
Source: Materials (Basel). 2026 Oct 19;19(20):4398. doi: 10.3390/ma19204398 (PMC13706813; doi:10.3390/ma19204398)
Supplement: Supplementary file 1 [file materials-19-04398-s001.zip › materials-4616083-supplementary.pdf]

Supplementary Information

Multiscale Degradation Behavior and Moisture State-Dependent Mechanisms of Solidified Slurry Under Engineering Service Conditions

This file includes: supplementary Figures S1 to S2.

The particle size distributions of the raw materials were measured using a laser particle size analyzer. The results are shown in S1.

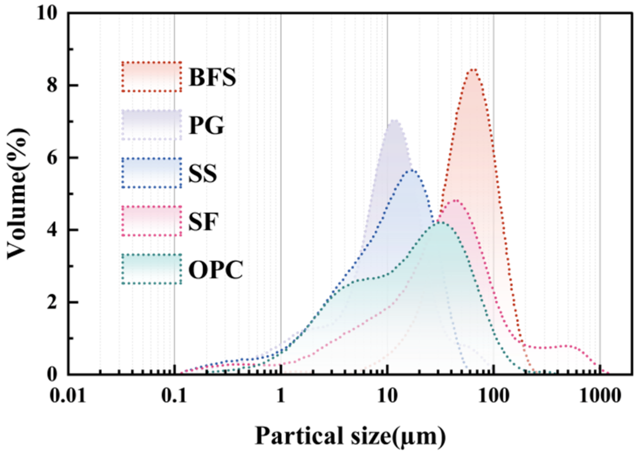

S1. Particle size distributions of the raw materials.

The overall experimental scheme is illustrated in S2.

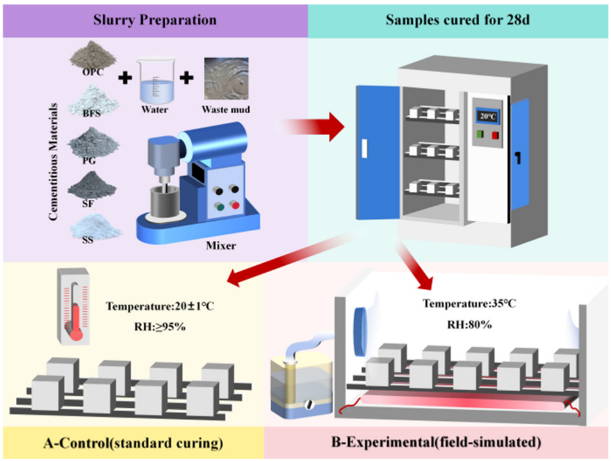

S2. Schematic illustration of specimen preparation and experimental design under different service conditions
